# Intermediate, Wholistic Shape Representation in Object Recognition: A Pre-Attentive Stage of Processing?

**DOI:** 10.3389/fnhum.2021.761174

**Published:** 2021-12-23

**Authors:** Jarrod Hollis, Glyn W. Humphreys, Peter M. Allen

**Affiliations:** ^1^Vision and Hearing Sciences Research Centre, Anglia Ruskin University, Cambridge, United Kingdom; ^2^Department of Experimental Psychology, University of Oxford, Oxford, United Kingdom

**Keywords:** wholistic, shape, object, attention, intermediate, vision

## Abstract

Evidence is presented for intermediate, wholistic visual representations of objects and non-objects that are computed online and independent of visual attention. Short-term visual priming was examined between visually similar shapes, with targets either falling at the (valid) location cued by primes or at another (invalid) location. Object decision latencies were facilitated when the overall shapes of the stimuli were similar irrespective of whether the location of the prime was valid or invalid, with the effects being equally large for object and non-object targets. In addition, the effects were based on the overall outlines of the stimuli and low spatial frequency components, not on local parts. In conclusion, wholistic shape representations based on outline form, are rapidly computed online during object recognition. Moreover, activation of common wholistic shape representations prime the processing of subsequent objects and non-objects irrespective of whether they appear at attended or unattended locations. Rapid derivation of wholistic form provides a key intermediate stage of object recognition.

## Introduction

Visual object recognition involves a series of stages in which different representations of object form are coded. Typically, it is assumed that early stages of vision involve the independent coding of local visual elements followed by a grouping of those elements into the part and whole representations of objects (Julész, 1971, 1975, 1981a,b, 1984; Julész et al., 1973, 1978; Julész and Bergen, 1983; Kim and Biederman, 2012). Theories differ however in terms of the emphasis they place upon part vs. the whole coding, and on whether parts may be derived independently of and in parallel with the coding of perceptual wholes (Biederman, 1987a,b; Biederman and Cooper, 1991, 1992; Hummel, 1992, 2000, 2001; Fenske et al., 1996; Hummel and Stankiewicz, 1996; Bar, 2003; Hayworth and Biederman, 2006).

The role of attention is to bind visual information and occurs early during visual analysis. The Feature Integration Theory (FIT) proposed by Treisman and Gelade (1980) accounts for the binding of elements with visual attention determining which features are bound at specific spatial locations. Much of the behavioural evidence for FIT has been derived from visual search experiments (Treisman and Gelade, 1980; Treisman, 1985). In these classic FIT experiments, the style of the search was manipulated to determine the allocation of attention by manipulating the search criteria.

An alternative approach has been proposed by Duncan (1984) who asserts that attention is directed to objects rather than spatial positions. Duncan and Humphreys (1989) presented evidence that similarity of the target and distractors can influence the style of perceptual processing that occurs. They argue that a conjunction style search requires binding and spatial attention, whereas a feature search does not.

The exact time course of binding is ambiguous, and the definition of what may be classified as pre-attentive is also a subject of debate. Ware (2004) suggested that there are four stimulus properties that elicit pre-attentive processing: these being: colour, form, movement, and spatial. He asserts that pre-attentive stages of vision occur up to ~500 ms post-stimulus presentation. Other definitions would claim pre-attentive defines a more non-conscious awareness of a shorter duration (Marcel, 1983). For these studies, the term pre-attentive will be used to denote the cognitive processing of a stimulus that occurs with limited awareness prior to attention being deliberately moved to a particular stimulus or location. Pre-attentive processing is thought to identify basic stimulus features in parallel, with high capacity (Posner, 1980; Treisman, 1985, 1991; Treisman and Souther, 1986; Treisman and Gormican, 1988; Posner and Petersen, 1990).

Theories have also considered the extent to which top-down factors influence objects at a post-perceptual level of representation. The role that attention plays in selection is a topic of debate. Some studies demonstrate that task-irrelevant material cannot be entirely overridden by top-down control, whilst others have suggested that material outside of the focus cannot capture attention (Santangelo et al., 2007; Botta et al., 2010, 2011). However, Theeuwes (1991, 2004) has pointed out that the attentional beam may operate as a zoom lens, and therefore the attentional capacity does not necessarily have a fixed dimension.

One approach to examining the different stages of object recognition is to evaluate priming effects from one object onto another. In an influential series of experiments (Biederman and Cooper, 1991, 1992, 2009a,b,c; Biederman and Gerhardstein, 1993, 1995; Fiser et al., 1996; Bar and Biederman, 1999; Biederman and Bar, 1999; Amira et al., 2012) assessed whether the presentation of parts of objects could prime the subsequent naming of the same objects. They found that object naming was primed from the presentation of parts of objects, and that the priming effects did not depend on the same exact contours being present in the prime and target stimuli. Biederman and Cooper (1991) argued that priming was dependent on the initial stimulus activating a common object representation from component representations of the parts of an object.

This account of a parts-based and view-independent recognition process contrasts with other arguments. Hummel and Stankiewicz (1996), for example, proposed a hybrid account in which parts-based, view-invariant coding operates alongside a more wholistic, view-specific process. They suggest that the parts-based approach is utilised when participants can devote processing resources to the stimuli, but that, when stimuli are unattended, then only the wholistic, view-specific process is used. Stankiewicz et al. (1998) also used priming procedures to support their argument and considered whether priming occurred for reflected images at either attended or unattended spatial locations. Attended primes facilitated subsequent target recognition in both the same view and reflected view conditions, whereas ignored primes produced facilitation only for primes presented in the same view as the target. These results suggest that the effects of attention (attended vs. not attended) and view (same vs. reflected) are additive in nature, i.e., when visual processing involves active attention, recognition is invariant to reflection. Hybrid accounts have received support from several sources including brain imaging using fMRI adaptation procedures that mimic priming, which has identified regions responsive to both view-specific and view-invariant representations of objects (e.g., Reisenhuber and Pogio, 1992; Logothetis et al., 1995; Logothetis and Sheinberg, 1996; Kourtzi and Kanwisher, 2000; Grill-Spector et al., 2001; Vuilleumier et al., 2001, 2002; Orban et al., 2004; van Boxtel et al., 2010; Thoma and Henson, 2011). Vuilleumier et al. (2002) found that regions in the ventral visual stream centre around the fusiform gyrus in the left hemisphere show adaptation both when objects have the same and different views across repetitions, while homologue regions in the right hemisphere show only view-specific adaptation. The study by Thoma and Henson (2011) utilised fMRI with an experimental manipulation that included a comparison of split and intact object images. This aimed to negate the influence of view-based representations. The right intra-parietal region showed repetition enhancement for intact primes. In contrast, fMRI repetition suppression was found in the left mid-fusiform region but only for attended primes. These data suggest that the left ventral stream performs analytical processing that requires attention, whereas, regions of the dorsal stream perform more holistic processing independent of attention.

The experiments reported by Hummel and Stankiewicz (1996) emphasised whether recognition was view-invariant or not rather than whether the underlying representation was parts-based or more wholistic in nature. Other authors, however, have argued for a rapid but wholistic process, which can provide an initial “scaffold” for subsequent recognition processes. For example, Parker et al. (1996) presented participants with either (i) low-spatial frequency components of objects (capturing overall, wholistic object properties) prior to high-spatial-frequency components (containing detailed information about object parts) or (ii) high-spatial frequency components before low-spatial frequency representations of objects. They found superior identification when low-spatial frequency components came first and argued that this reflected a natural order in which the coding of rapidly derived low-spatial frequency representations of objects preceded the coding of more slowly derived high spatial frequency representations. Similarly, Bar (2003) has argued for the rapid coding of low-spatial frequency representations which he argues are used by frontal lobe structures to form “hypotheses” of the objects being coded. These hypotheses act to guide a slower-acting parts-based recognition process. In a similar proposal, Fenske et al. (1996) suggested that early visual selection is guided by low spatial frequency information, and this coarse coding is then refined using top-down and contextual information.

A second approach to understanding the representations sub-serving object recognition is to assess the breakdown of the process in brain-lesioned patients, where selective damage may reveal the nature of the underlying processes. There is strong evidence that damage to parts-based recognition processes can disrupt object identification. For example, Humphreys et al. have reported analyses of an agnosic patient, HJA, who had severe problems in parts-based object recognition, for example, is disrupted when visual elements were present but overlapping each other, i.e., when the parts were available but not the overall shape. They proposed that damage to the processes that normally group component parts of objects led to difficulties. Interestingly, however, HJA was better able to identify silhouettes than line drawings of objects (Riddoch and Humphreys, 1987; Humphreys et al., 1988), suggesting that he could access stored knowledge from the overall shape. HJA also showed global shape advantages when required to identify compound hierarchical shapes (cf. Navon, 1977; Humphreys and Riddoch, 1987; Riddoch et al., 2008). This global advantage can be attributed to rapid access to low-spatial frequency representations of objects, even though these representations are not sufficient to generate accurate recognition for all objects. Lestou et al. (2014) also report that HJA showed the normal perception of global form and that this process was likely mediated by regions of the parietal cortex responsive to global form information. In contrast, MH, a patient with a damaged parietal cortex but structurally and functionally intact ventral cortex was impaired at perceiving the global forms. Such results suggest that global representations of objects may be derived in parallel with more parts-based analyses, which normally integrate with the wholistic representations to enable recognition of individuated objects to take place. In patients, such as, HJA, this integration process is damaged but global shape perception remains possible.

Neuropsychological evidence also indicates that, in the absence of attention, recognition may be view-specific rather than view-invariant (Lawson et al., 1994; Tarr et al., 1998; Ullman, 1998; Schill et al., 2020). Vernier and Humphreys (2006) examined patients with unilateral neglect after damage to their parietal cortex who were poor at attending to stimuli in their contra-lesional field. These patients showed impaired matching and showed no priming between mirror-reflected objects, even though the patients could match part object representations presented to their contra-lesional field. Like Stankiewicz et al. (1998), Vernier and Humphreys proposed that attention to the contra-lesional side of objects was necessary to create robust view-invariant object representations.

If spatial attention was found to interact with the priming of shape, that would suggest that a common stage of processing was affected. Alternatively, it may indicate that the effects of spatial attention and structural priming were additive which would be consistent with attention and priming affecting independent processing stages. In all the experiments critical primes were structurally rather than conceptually related to targets, and the nature of the structural relations (e.g., the stimuli could share outline forms or component parts) were varied. Was structural priming greater for stimuli that were related in terms of their overall form rather than their parts? Priming on objects (stimuli with stored representations) and non-objects (lacking stored representations) were also examined. Was priming greater for objects due to the role of activated stored knowledge? or was priming equally effective for objects and non-objects?. Note that this would be consistent with priming based on the activation of intermediate representations constructed for the processing of objects and non-objects alike.

In this article, we report novel evidence for the pre-attentive coding of wholistic shape representations in object processing using data from priming effects in normal observers. In this series of experiments, participants viewed a briefly presented prime object which was followed by another object or non-object and participants had to make an object decision concerning the target. The prime and target could appear at the same location so that the prime validly cued the spatial location of the target (on valid trials) or the prime and target could appear at different locations (on invalid trials). The aim was to determine whether any structural priming from the initial stimulus (based on its overall shape or its component parts) was enhanced when the target fell at an attended location relative to when it fell at an unattended location (cf. Posner, 1980). It was hypothesised that valid trials would benefit from the direction of gaze, as attention would already be located where the target would appear. This assumption concurs with the literature concerning inhibition of return (Klein, 2000; Lupiáñez et al., 2006).

Experiment 1 aimed to determine whether priming would occur from the similarity in visual structure, and how the magnitude of this priming would compare to conceptual priming. A cued paradigm would be used to determine whether there was an effect of attention. Experiment 2 assessed whether these priming effects occurred due to parts that were in common between prime and target stimuli or whether it was the similarity of the outline shape that was critical. In addition, effects were explored with both object and non-object targets, to evaluate if priming depended on activation of a stored representation of targets. Experiment 3 varied whether the primes were whole outlines, edges, or edge fragments. Experiment 4 assessed priming from whole shapes and shape components. Experiment 5 considered whether the effects of priming would occur when attention was directed using a dual-task. Experiment 6 considered whether priming would occur following a very brief exposure when the prime was outside conscious awareness. Finally, Experiment 7 tested whether priming depended on particular spatial frequency components in primes.

## Experiment 1: Structural Priming

The first experiment considered whether an object image would prime recognition of a similar-looking but unrelated object image. In prior work, structural priming between objects has been examined in several ways. Biederman et al. have examined structure-based priming and argued that priming can reflect parts-based access to a common object representation (when non-accidental properties in each parts-based image enable the common representation to be coded) (Biederman, 1987a,b, 2007; Hummel and Biederman, 1992; see above). Boucart et al. (1995) examined structural priming between different, visually similar objects and reported that visually similar objects could prime one another, even when the initial primes were briefly presented and masked. Thus, suggesting that it is unlikely that priming was based on participants using a conscious strategy of matching common elements in similar stimuli. Here, we extended these original experiments by Boucart et al. (1995) to assess the nature of the visual representations mediating the effects and whether the effects were dependent on attending to the locations of the stimuli.

### Method

#### Design

A factorial design was used with Cue, Prime, and Interval all being within-participant factors. The factor of Cue had two levels: valid cues were trials in which the prime and target appeared in the same location, and invalid cues were trials in which the prime appeared in a different location to the target. The factor of Prime had four levels: structurally related trials were where the prime-target pair shared a visually similar structure, but were not associated or conceptually related; conceptually related trials were where the prime-target pair came from the same semantic category, but they were not associates and they were visually dissimilar; neutral trials where a simple shape was presented as a prime; and unrelated trials where the prime-target pairs were re-assigned so that there was no relationship between the items on a trial. The factor of Interval had two levels: the inter-stimulus interval (ISI) between the prime and the target was either 200 or 500 ms (short or long ISI).

#### Apparatus and Stimuli

**Figure 1:**
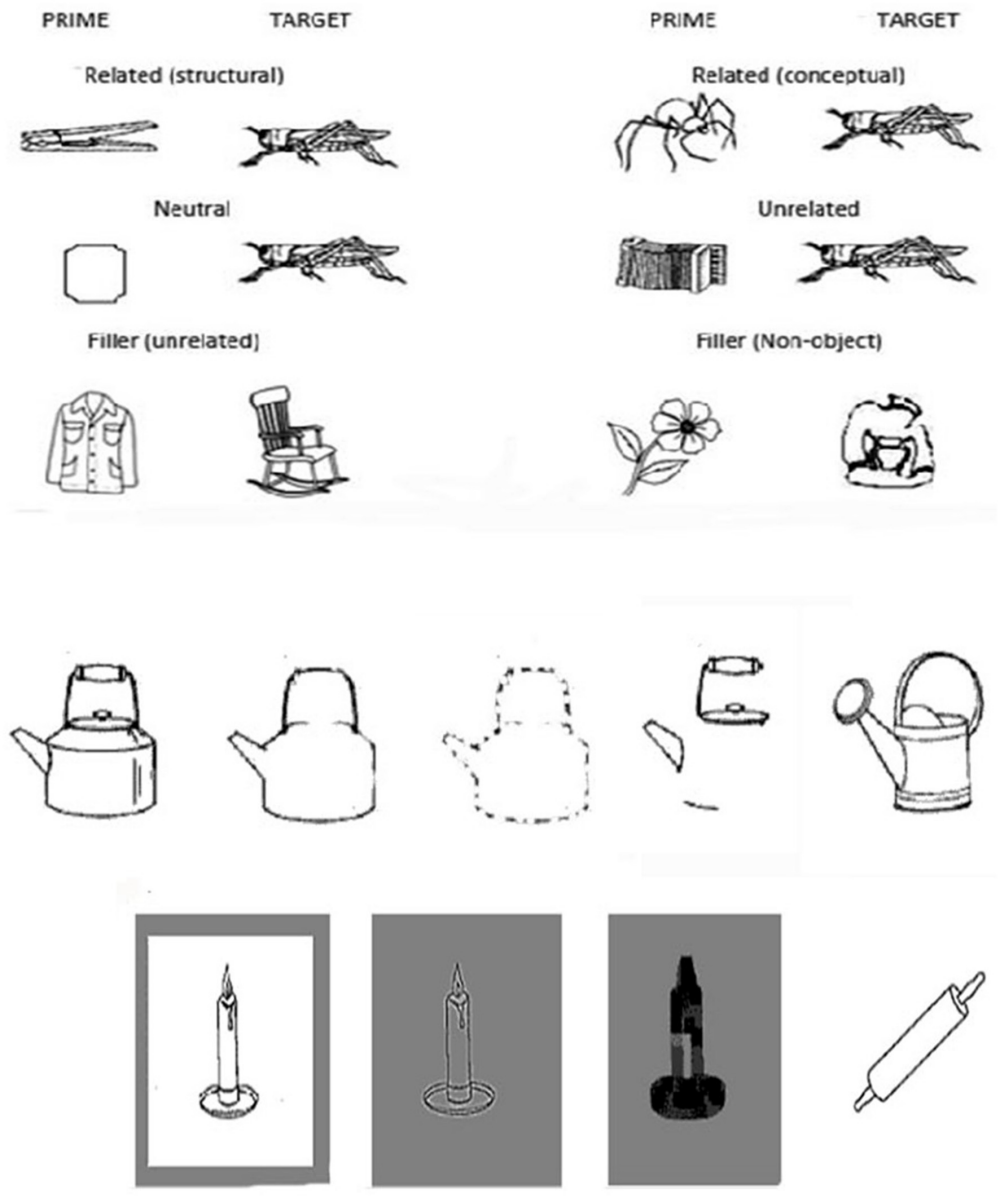
Examples of the experimental pairings from experiment 1 (top figure). Examples of whole, shape, fragment, and components primes and target from experiments 2–6 (middle figure). An example of the high spatial frequency and low spatial frequency primes and target from experiment 7 (bottom figure).

Line drawings of objects from Snodgrass and Vanderwart (1980) were arranged into pairs so that each target had a structurally similar prime and a conceptually similar prime. Unrelated primes were formed by reforming the pairs, and neutral primes were formed with a set of simple object shapes. A set of non-object images was taken from Kroll and Potter (1984) to form a series of filler items in which the primes were unrelated object images that were not used elsewhere. An example of the object pairings is shown in Figure 1.

There were 240 experimental trials, half of which required a positive response to the target and half required a negative response to the target (i.e., 120 experimental trials and 120 filler trials). The 120 experimental trials were divided as follows. There were 66 pairings for valid cues: 22 related pairings (11 structurally related pairs and 11 conceptually related pairs), 22 neutral pairings, and 22 unrelated pairings. There were 54 pairings following invalid cues: 18 were related pairs (9 structurally related and 9 conceptually related), 18 pairs were neutral, and 18 were unrelated. The duration of the prime was 200 ms. Each trial was repeated once with a short ISI (200 ms) and once with a long ISI (500 ms). The conditions were randomly ordered for each participant and the targets in the different conditions were counterbalanced across participants. The experimental trials were preceded by one block of 12 practise trials. The practise items did not appear in the experimental pairings.

The stimuli were selected based on ratings. Using a Likert scale 1–10, 10 participants not involved in the experiments were asked to rate how visually similar pairs of items were. These pairings were grouped to use as experimental stimuli. The pairings classed as structurally related had an average similarity rating of 7.1 (*SD* = 0.8), the conceptually similar pairs had an average similarity rating of 2.1 (*SD* = 0.7) and the unrelated pairs had a mean similarity rating of 1.3 (*SD* = 0.3) Similarity ratings between structural and conceptual primes [*t*_(38)_ = −27.30, *p* = 0.0001] and the structural and unrelated primes [*t*_(38)_ = 31.30, *p* = 0.0001] were significantly different. Similarity ratings between conceptual and unrelated primes were not significant [*t*_(38)_ = 1.32, *p* = 0.19]. These comparisons confirmed that the structurally similar pairs were highly similar in appearance, but the conceptually and unrelated pairings were not.

Line drawing stimuli were sized to fit into a 2.5 cm square area. The viewing distance was 75 cm from a screen giving a visual angle of ~2° per stimulus. There was a central fixation and stimuli appeared 4 cm (3°) on either side of the central fixation. The prime presentation also acted as a spatial cue. On valid trials, the prime and target appeared in the same place whereas, for invalid trials, the prime and target appeared on the opposite side of central fixation separated by a visual angle of 6°. The valid trials would therefore benefit from the direction of gaze, as attention is automatically directed to an area of peripheral visual stimulation (Hunt and Cavanagh, 2009).

**Figure 2:**
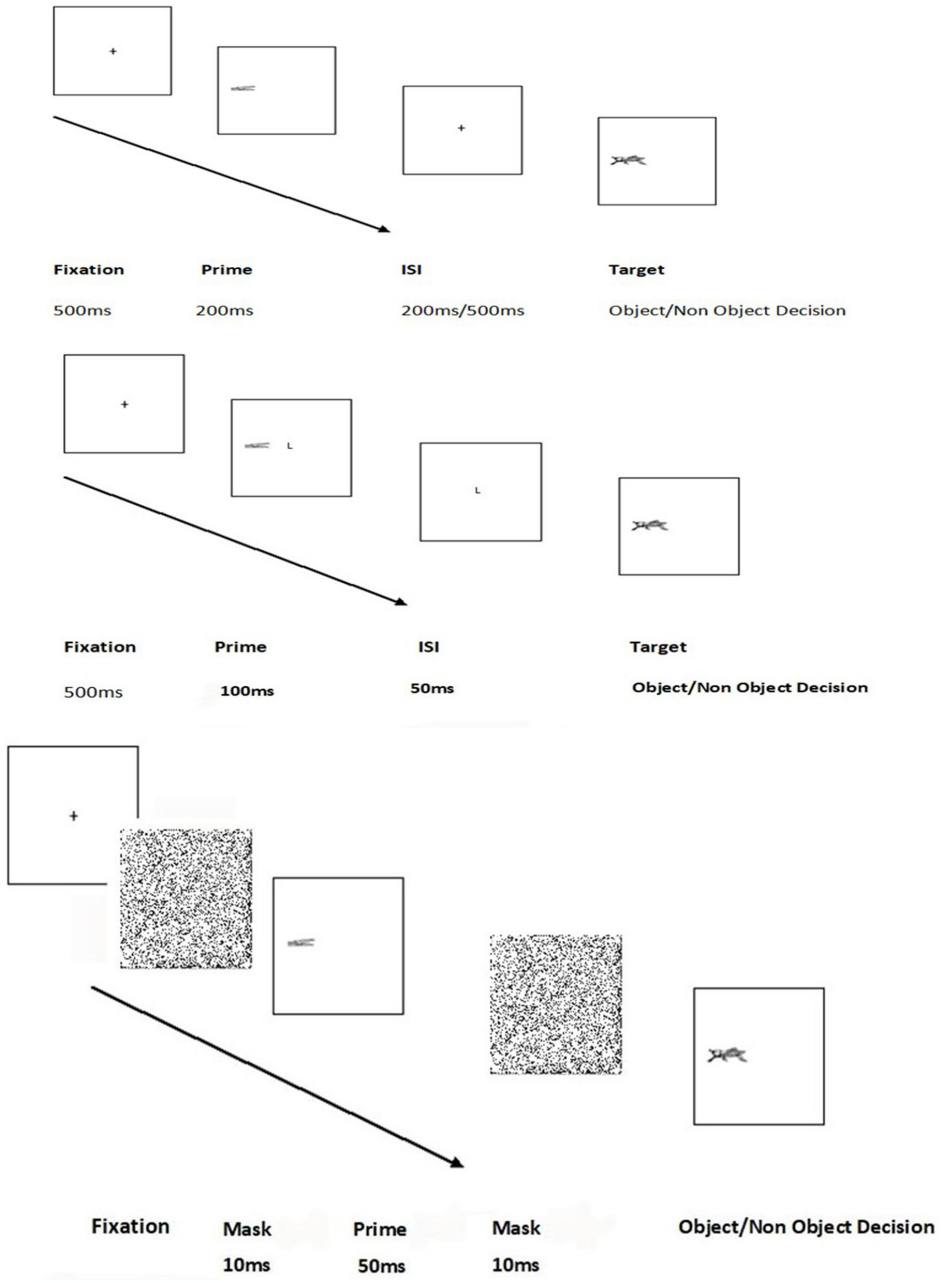
Trial sequence for experiments 1–4 and 7 (top figure) experiment 5 middle figure) and experiment 6 (bottom figure).

Each trial consisted of a fixation, followed by a prime (duration 200 ms) that appeared to the left or right of fixation. Subsequently, the screen cleared and following an interval of 200 or 500 ms the target appeared, in the same position (valid trials) or the opposite side (invalid trials). The target remained on the screen until the participant made a decision. The experiment was presented on a PC using Superlab Pro 2.04 software (Cedrus Corporation, CA, USA). Responses were taken through keypress. The trial sequence is illustrated in Figure 2.

#### Participants

Power analysis conducted using G^*^power (University of Düsseldorf, Germany) suggested that for a within-participants ANOVA of three comparisons a sample of 18 would be needed to obtain statistical power at the recommended.8 level (Cohen, 1988).

An opportunity sample of 20 adult participants (12 female, 8 male) from the Kings Lynn Vision Plus Ltd (Norfolk, UK). volunteered to take part in the experiment. Age was not an inclusion criterion apart from participants being over 18 years old. This was an adult workforce, rather than a student sample, and therefore resulted in a wide age range. The ages ranged from 20–64 years (mean 36 years, *SD* = 9).

#### Procedure

Participants were told that they would be conducting an experiment about real and unreal object images. They would see target object images that occurred shortly after a prime and the task was to make an object decision (real object vs. unreal object) to the target as quickly and accurately as possible. They were told that the prime provided information about where the next item would appear. Each participant received 12 practise trials followed by 480 experimental trials.

### Results

#### Reaction Times

**Figure 3:**
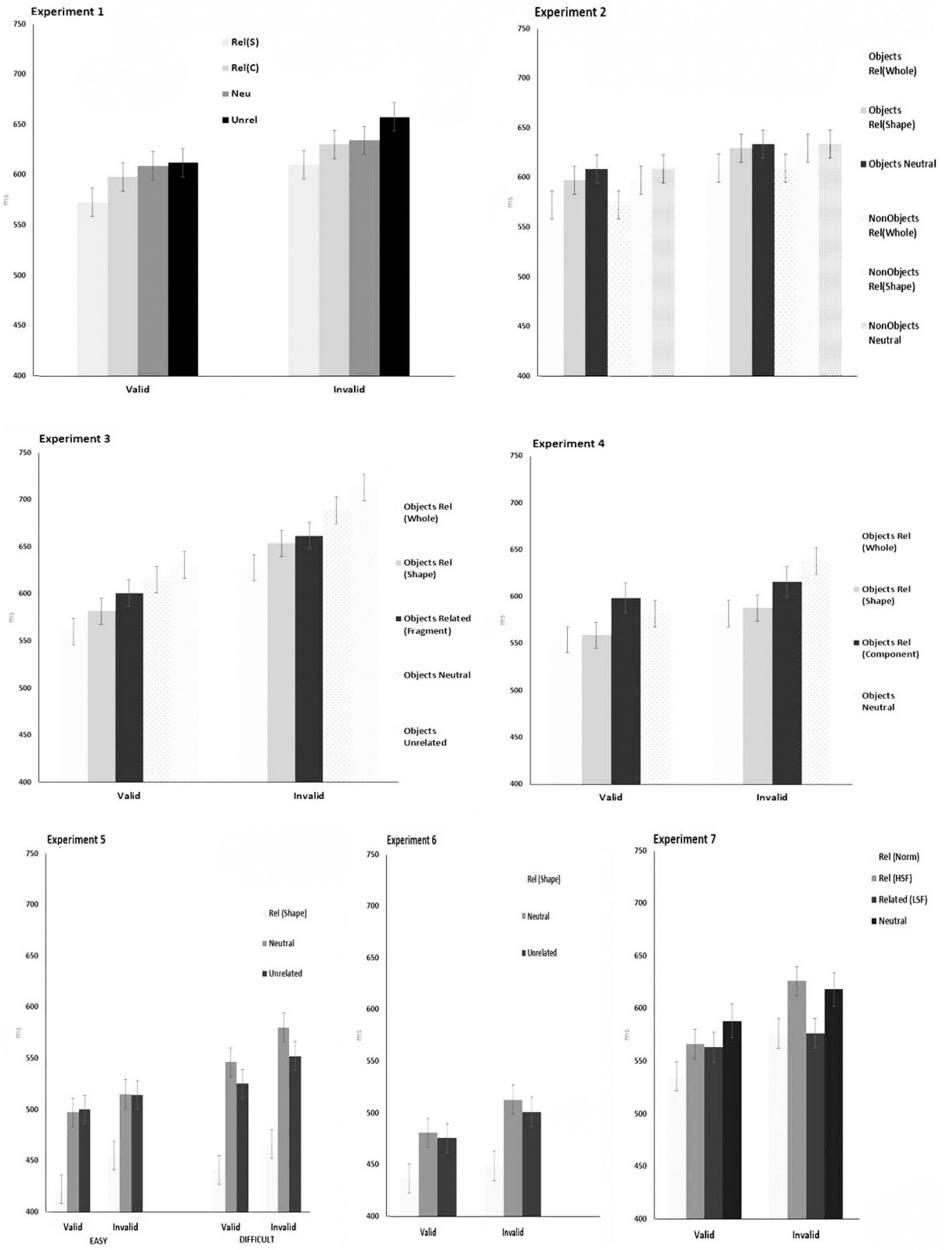
Mean RT for experiments 1–7. Error bars illustrate the 95% CI for cue × relation.

Throughout the series of experiments, only data from correct responses were subjected to analysis. Data points of more than 3 *SDs* from each participant's mean were treated as errors. For Experiment 1, this accounted for <1% of the total responses. The mean correct RTs and percentage errors are illustrated in Figure 3.

A *post-hoc* analysis of achieved power was determined using G-Power. For the sample of 20 participants, the critical value of *F* was 2.79 and the effect size for the *F*-test was 0.8. This resulted in a power of 98%. The use of such analyses has been subject to criticism. Levine and Ensom (2001) asserts the 95% CI may be a more appropriate method of determining statistical power. We have therefore included the 95% CI on all of our figures (Loftus and Masson, 1994; Cousineau, 2005).

Response times were subjected to ANOVA. There was a significant main effect of Cue *F*_(1,18)_ = 29.38, *p* < 0.0001. There was a significant main effect of Prime *F*_(3,54)_ = 9.67, *p* < 0.0001. The main effect of Interval was not significant *F*_(1,18)_ = 1.15, *p* = 0.29. Pairwise comparisons were carried out on the effect of Prime using Student-Newmann-Keuls. Significant differences were found between neutral and structurally related primes (mean diff: 20.98, crit diff 17.95, and *p* < 0.05) neutral and unrelated primes (mean diff: −18.91, crit diff: 14.95, and *p* < 0.05) unrelated and structurally related primes (mean diff: −39.9, crit diff: 19.90, and *p* < 0.05) and related conceptual and neutral primes (mean diff: −22.69, crit diff: 17.95, and *p* < 0.05). The difference between the neutral and conceptual primes did not reach significance (mean diff: 3.77, crit diff: 14.95, and *p* < 1). None of the interactions reached significance (All *F*s < 1.6, All *p*s < 0.2). The mean RTs with 95% CI are illustrated in Figure 3.

#### Errors

The percentage of errors was lowest for the unrelated presentations followed by related than neutral presentations (related 2.6%, unrelated 1.5%, and neutral 3.8%). There was a significant main effect of Cue *F*_(1,19)_ = 30.03, *p* < 0.001. The effect of Prime did not reach significance *F*_(3,19)_ = 1.09 and *p* = 0.35. The main effect of Interval was not significant *F*_(1,19)_ < 1. The interaction between Cue and Interval was significant *F*_(1,19)_ =5.17, and *p* < 0.05. Other interactions did not reach significance (All *F*s < 1.5, All *p*s < 0.21).

### Discussion

Experiment 1 found evidence of priming of visual form between structurally related but different objects. The significant effect of the cue in both the RTs and error analysis demonstrated that there was an attentional component to the processing. The structural priming was strongest when the interval between the prime and target was short (200 ms) but reduced when the interval was increased to 500 ms. The pairwise comparisons carried out on the effect of Prime found significant differences between neutral and structurally related primes, but not between the neutral and conceptual primes. This priming effect cannot be attributed to the conceptual similarity between the structurally related primes and targets as the structurally related pairs came from different categories and were not associated.

Moreover, there was no evidence for conceptual priming when items were structurally unrelated but drawn from the same category. The reduced priming in the conceptual condition may reflect the distant semantic relations between the items; nevertheless, concept-level priming should still have been more evident than in the structurally related condition where there was no conceptual information in common. These data suggest that priming here was based on the visual similarity of the prime and target.

## Experiment 2: Priming Wholes and Outlines on Object and Non-Object Targets

Experiment 2 compared the priming effects found between visually similar objects and visually similar non-objects. Since the non-objects did not have stored representations, similar priming for the object and non-object pairs would suggest effects at intermediate levels of representation, prior to stored object representations being contacted. Differences between whole primes (containing both outline shape and local parts) and outline primes (only containing the outline shape) were also assessed to investigate whether the presence of internal parts was critical to generate priming.

### Method

#### Design

Experiment 2 used a 2 × 3 × 2 within-participants factorial design. The factor of Cue had two levels (valid, invalid); Prime had three levels (whole stimulus, outline shape, and neutral) and the factor of Target had two levels (object and non-object).

#### Stimuli and Materials

Primes were selected to be visually similar shaped objects to targets or neutral shapes. The visually similar shapes were then divided into two sets. In one case, the prime contained both the external and internal details of the stimulus. In the second, the prime was based on just the outline shape from the original whole stimulus. To verify that the selected items were highly similar, each pairing was then rated by 20 independent participants (8 men, 12 women) who had not taken part in the other experiments. Participants were asked to rate each pairing in terms of (a) the similarity of the overall shapes, and (b) the similarity of the internal features. A ten-point scale was employed with (1) representing “No similarity” and (10) representing “Identical similarity.” The pairs with the highest shape ratings were selected for use in the Experiments. The similar object pairs had a mean similarity rating of 5.8 (*SD* = 0.4) and the similar non-object pairs had a mean of 6.1 (*SD* = 0.3). The similarity of the objects and non-objects selected for this experiment did not differ (*t* < 1).

The shape primes were created by photoshop adaptation, so that they contained only the outline of the original stimulus image (refer Figure 1 for examples).

Both object and non-object targets included filler prime trials as well as trials with similar (whole and outline) and neutral primes. On these filler trials either an unrelated object preceded a non-object target, or an unrelated non-object preceded an object target. The trial sequence is illustrated in Figure 2. There was a total of 448 trials: 224 trials with objects as the experimental items and another 224 trials with non-objects as the experimental items. These trials consisted of 128 valid trials: 16 pairings for each condition with 16 fillers requiring a positive response, and 64 fillers requiring a negative response; 96 invalid trials: 12 pairings for each condition with 12 fillers requiring a positive response, and 48 fillers requiring a negative response. As in previous experiments, trials were randomly presented, and participants made the same object decision to the targets through keypress.

#### Participants

The participants were the same as Experiment 1.

### Results

#### Reaction Times

The mean correct RTs are illustrated in Figure 3. Data points of more than 3 SDs from each participant's mean were treated as errors. For Experiment 2, this accounted for <1% of the total responses. The data were explored with a repeated-measure ANOVA. There was a significant main effect of Cue [*F*_(1,19)_ = 98.60, *p* < 0.0001] indicating that valid cues produced faster responses than invalid cues. There was also a significant main effect of Prime [*F*_(2,38)_ = 10.62, *p* < 0.002]. The difference between whole primes and outline primes was not significant (mean diff: 9.97, crit diff: 13.96, and *p* < 1). In contrast, RTs on neutral prime trials were slowed relative to when whole primes appeared (mean diff: −31.10, crit diff: 16.80, and *p* < 0.05) and relative to when there were outline primes (mean diff: −21.13, crit diff: 13.96, and *p* < 0.05). There was also a significant main effect of Target [*F*_(1,19)_ = 41.20, and *p* < 0.0001], indicating RTs were slower to non-objects (mean: 651 ms, *SD* = 30.6) than object (mean: 607 ms, *SD* = 36) targets. None of the interactions reached significance [all *F*s > 0.13, all *p*s > 0.17].

#### Errors

For targets depicting real objects, the percentage of errors was lower for the valid presentations and higher for the invalid presentations (valid, 3%; and invalid 4.9%). Related presentations produced fewer errors than neutral presentations (related wholes, 2.9% related shapes 2%, and neutral 3.3%). For targets depicting non-objects, the percentage of errors was lower for the valid presentations and higher for the invalid presentations (valid, 3% and invalid 4.9%) Related presentations produced fewer errors than neutral presentations (related wholes, 3%, related shapes 2.9%, and neutral 3.1%).

There were reliable main effects of Cue [*F*_(1,19)_ = 4.5, *p* = 0.04] and prime [*F*_(2,38)_ = 4.22, *p* = 0.02] and a trend for a main effect of Target type [*F*_(1,19)_ < 0.06]. The 3-way interaction Cue × Prime × Target was also significant [*F*_(2,38)_ = 3.19, *p* < 0.05]. There was an increased error rate to non-objects compared with objects and this was particularly the case for neutral and filler priming trials.

### Discussion

Structural priming was again found in Experiment 2, and in this case, did not differ between whole primes (containing both the outline shape and the internal parts) and outline shapes. The significant effect of the cue demonstrated that there was an attentional component to the processing. This suggests that structural priming can be generated based on the similarity of the outline shapes of stimuli and that internal part-based information does not add significantly to the priming effect. The interactive component for the RT analysis was not significant. In contrast, a significant 3-way interaction amongst cue, prime, and target was found in the error analysis. These differences may have occurred because of the change in timing and the different number of experimental trials from Experiment 1. This interpretation will be addressed in later experiments where the timing is manipulated. The 3-way interaction may also indicate that the processing of the prime has an attentional component that may depend on the nature of the stimuli. How well this conclusion holds will be tested in the subsequent experiments.

One other critical result from Experiment 2 was that structural priming was as strong on non-object targets as on object targets. This result strongly suggests that priming here was not dependent on primes activating a stored representation that is also accessed by the target, so facilitating the recognition of familiar object targets. If this were the case, then there should not have been priming for non-objects. These data suggest that an intermediate representation, common to both objects and non-objects, is coded from the prime, and there is facilitated processing of targets with a similar intermediate representation.

## Experiment 3: Intact vs. Fragmented Outlines

The aim of Experiment 3 was to determine whether particular aspects of the visual structures of primes were responsible for the priming effects. We contrasted priming from similar whole objects, outline object shapes (as in Experiment 2), and where the outline shapes were fragmented into distinct contours.

### Method

Unless otherwise mentioned the method was the same as in Experiment 2. The object images from Experiment 2 were used again and the whole primes (and the associated similarity ratings) were the same as before. The image pairs were then adapted so that in one condition only a single continuous outline remained without any visual information about internal parts. In an additional condition, the contour was fragmented into parts. The fragmented contours were formed by taking the outline shape and then randomly erasing 50% of the pixels from the contour, whilst, preserving any junctions or significant regions (see Figure 1).

There was a total of 168 trials (48 valid trials: 16 pairings for each condition with 48 fillers and a corresponding 36 invalid trials with 36 fillers). The trial sequence is illustrated in Figure 2. As in previous experiments, participants received a series of practise trials prior to the experimental presentation, and the task was to make an object decision to the target objects and non-objects through keypress.

#### Participants

The participants were the same as Experiments 1 and 2.

### Results

#### Reaction Times

The mean correct RTs are illustrated in Figure 3. Data points of more than 3 *SD*s from each participant's mean were treated as errors (<0.5% of the total responses). RTs were subjected to a repeated-measure ANOVA with the factors being Cue and Prime. Responses to targets that followed a valid cue (mean 596 ms) were significantly faster than those following an invalid cue (mean 669 ms) [*F*_(1,17)_ = 53.98, and *p* < 0.001]. There was also a significant effect of Prime [*F*_(4,68)_ = 20.54, and *p* < 0.001]. The difference between shape primes and fragmented primes (mean diff: −13.94, crit diff: 20.47, and *p* > 0.05 was not significant. The other comparisons all produced significant differences at the 0.05 level of significance. The interaction between cue and priming was not significant [*F*_(4,68)_ < 1].

#### Errors

A similar ANOVA to that performed on the RT data failed to reveal any significant effects (all *F*s < 3.24; all *p*s > 0.095).

### Discussion

Structural priming in Experiment 2 was found both when whole objects were primed and when just the outline shape of the same objects was used (as in Experiment 3). The priming effect was not reliable when the outline edge was fragmented, where the data fell between that observed with outline shapes and neutral primes. However, prior work on the recognition of line drawings has shown that the way in which images are fragmented can make an enormous difference to how easily they access stored knowledge–fragmentation that leaves intact non-accidental features of objects has a minimal effect on recognition whilst recognition suffers greatly if non-accidental features are not preserved (Biederman, 1987a,b).

## Experiment 4: Matching for Contour

In this study, the stimuli from Experiments 2 and 3 were reformed to include the whole primes, outline shape primes, and component (feature) primes. Examples of the stimuli can be found in Figure 1. The feature primes were selected to enable the object to be identified (i.e., the objects were recoverable) and to be matched in terms of the amount of contour to the outline shape primes. If the amount of contour is critical, then priming in the outline and component feature conditions should be matched.

### Method

The method was the same as in Experiment 3. A count of high contrast pixels was taken from each prime, and the shape and component primes were adjusted so that there was an equivalent number of high contrast pixels in each. The whole primes had an average of 722 high contrast pixels, and the outline shape and component primes had averages of 411 and 445 pixels, respectively. There was no significant difference between the number of pixels in the outline shape and component conditions, *F*_(1,15)_ < 1, *p* = 0.41. There were 4 priming conditions—where primes were whole objects, outline contours, component objects, and neutral shape primes. The numbers of stimuli per condition and the proportion of valid: invalid trials were kept the same as the previous experiments. The trial sequence is illustrated in Figure 2.

#### Participants

Twenty adult participants (10 female, 10 male) from Kings Lynn Vision Plus Ltd., volunteered to take part in the experiment. The ages ranged from 20 to 50 years (mean 44, *SD* = 8) and none had taken part in the previous experiments.

### Results

#### Reaction Times

Data points of more than 3 *SD*s from each participant's mean were treated as errors (<1% of the total responses). The mean correct RTs are Illustrated in Figure 3. There were significant effects of Cue [*F*_(1,19)_ = 13.88, *p* = 0.001] and Prime [*F*_(3,19)_ = 13.15, *p* = 0.001]. No significant differences were found between RTs following whole primes and outline shape primes (mean diff: −13.94, crit diff: 20.73, and *p* > 0.05). Whole primes and outline shape primes both facilitated performance relative to the component part and neutral shape conditions (whole vs. component parts: mean diff: −37.72, crit diff: 24.92, and *p* < 0.05; whole vs. neutral mean diff: −63.42, crit diff: 27.43, and *p* = 0.05). No other comparisons were significant. The interaction between Cue and Prime was not significant, *F*_(3,57)_ = 1.45, *p* = 0.24.

#### Errors

The effect of Prime was not significant, *F*_(3,19)_ < 1, *p* > 0.05 although there was a significant effect of Cue, *F* (1,19) = 11.08, *p* = 0.03. There were more errors following invalid trials (6%) than following valid trials (5.8%). The interaction between Prime and Cue was not significant *F*_(3,57)_ = 1.32 and *p* = 0.27.

### Discussion

Experiment 4 again demonstrated the effects of structural priming from a similar whole object shape, but not from object components. This result was confirmed using outline forms and component primes matched for their number of pixels, confirming that the presence of the global shape outline rather than the parts of objects was critical.

In the previous experiments, the prime presentation acted as both the prime and also a cue. The valid trials would benefit from saccadic eye movements and direction of gaze phenomenon whereas the invalid trials would not. Could this have resulted in the bias of the valid trials? Previous studies have shown that saccadic eye movements are generated more quickly when they concur with the direction of a forthcoming target (Wolohan and Crawford, 2012). Visual saccadic movements have an onset of approximately 200 ms, and therefore the timing of the previous experiments would have allowed the valid trials to benefit from the direction of gaze. To address these issues, the prime duration and interval were reduced to 100 ms and in addition, a dual-task presentation was used to control directed attention during the prime presentation. Potentially the lack of naiveness of the participants may have been an issue. This will be addressed in the forthcoming experiments, with a change of participants, and in experimental presentations that are too short or manipulated to negate the use of conscious strategies.

## Experiment 5: Priming of Outlines With Directed Attention

Experiment 5 considered the effect of attention on the prime task by manipulating directed attention. A dual-task approach was taken such that at the moment of the prime appearing, participants were required to perform a task at fixation. This would occupy directed attention and ensure that the prime occurred outside of the attentional beam.

Dual-task presentation has been used in previous research to investigate vision outside of the focus of attention (Braun and Sagi, 1990, 1991; Tapia et al., 2014). During the prime presentation, attention is focussed on a central task, whilst an experimental stimulus appears elsewhere in the visual field. In this way, the focus of awareness is controlled and can vary by manipulating the task demands of the item held in focus.

Previous research has shown that saccades depend on “the task set” is compatible with the saccade programme (Koval et al., 2005). Giving the participants a task at fixation during the prime presentation would help to ensure that the task set would impede saccades toward the prime.

### Method

The method was the same as in previous except that (a) the duration of the prime was reduced to 100 ms with an ISI between prime and target of 50 ms and (b) during the prime presentation the fixation was altered to appear as a coloured letter (either T or L appearing in red or green font). Participants were required to make a decision based on the fixation item, and verbally report their decision at the end of each trial. The difficulty of the decision was manipulated in two ways: For an easy task, participants were required to report the colour (red or green) of the fixation letter. For a harder task, participants were required to report the identity of the letter (either a T or an L). Participants were required to attain at least 75% accuracy in the fixation task in order for data to be included. There were 3 priming conditions, namely, related shape primes, neutral primes, and unrelated shape primes. As in the previous experiments, participants were asked to make an object decision for each target, by keypress using their dominant hand. Following their key response, the participants were then required to make their verbal reports. The numbers of stimuli per condition and the proportion of valid: invalid trials were kept the same as the previous experiments. The trail sequence is illustrated in Figure 2.

#### Participants

Twenty adult participants (12 male, 8 female) from Kings Lynn Vision Plus Ltd., volunteered to take part in the experiment. The ages ranged from 20 to 50 years (mean 48, *SD* = 8) and none had taken part in the previous experiments.

### Results

#### Reaction Times

The mean correct RTs are illustrated in Figure 3. As in the previous experiments, data points of more than 3 *SD*s from each participant's mean were treated as errors (<1% of the total responses). There was a significant main effect of Task [*F*_(1,19)_ = 4.08, *p* = 0.04]. There was a significant main effect of Priming [*F*_(2,38)_ = 148.2, *p* = 0.0001]. There was a significant main effect of Cue [*F*_(1,19)_ = 95.85, *p* = 0.0001]. The interactions between Task × Cue, Priming × Cue, and Task × Priming × Cue did not reach significance (All *F*s < 1.5, All *p*s < 0.22). There was a significant interaction between Task and Priming [*F*_(2,38)_ = 8.88 *p* = 0.0007]. The Task × Priming interaction was explored using separate ANOVA for the task, with contrasts of means using Student-Newmann-Keuls.

In respect of the easy task there was a significant effect of Priming [*F*_(2,19)_ = 47.84, *p* = 0.0001]. Comparisons of means showed that the was a significant difference between the Related vs. Neutral (mean diff: 67.6, crit diff: 20.1, and *p* = 0.001) and Related vs. Unrelated (mean diff: 68.8, crit diff: 20.1, and *p* = 0.001) but no significant differences were found between the Neutral vs. Unrelated conditions (mean diff:1.25, crit diff: 20.1, and *p* = 0.87 NS). There was a significant effect of Cue [*F*_(1,19)_ = 32.47, and *p* = 0.0001]. The Interaction between Relation and Cue was not significant [*F*_(2,38)_ = 2.79, *p* = 0.07].

For the hard task, there was a significant effect of Priming [*F*_(2,19)_ = 140.2, *p* = 0.0001]. Comparisons of means showed that the was a significant difference between the Related vs. Neutral (mean diff: 109, crit diff: 17.16, and *p* = 0.001) and Related vs. Unrelated (mean diff: 85.25, crit diff: 17.16, and *p* = 0.001) and there was significant difference between the Neutral vs. Unrelated conditions (mean diff: 23.95, crit diff: 20.1, and *p* = 0.01). There was a significant effect of Cue [*F*_(1,19)_ = 43.31, *p* = 0.0001]. The Interaction between Priming and Cue was not significant [*F*_(2,38)_, *p* < 1].

#### Errors

There was a significant main effect of Priming [*F*_(1,19)_ = 5.4, *p* = 0.006]. The percentage errors were higher in the Neutral and Unrelated conditions (8 and 4.3%, respectively), compared to the Related condition (3.1%). There was a significant main effect of Task [*F*_(2,38)_ = 5.22, *p* = 0.009]; More errors occurred with the hard task (mean 0.64, *SD* 0.92) than the easy task (mean 0.34, *SD* = 0.6). There was a significant main effect of Cue [*F*_(1,19)_ = 4.90, *p* = 0.03] indicating that fewer errors were found after the Valid trials (mean 0.4, *SD* = 0.71) compared to the Invalid trials (mean.58, *SD* = 0.86). None of the interactions reached significance [All *F*s < 2.1; All *p*s < 0.5].

### Discussion

The manipulation of attention using the dual-task presentation evoked similar priming to those of the previous experiments. Significant priming of shape was present, and this was found even when the prime presentation required participants to attend to the fixation as the prime image was presented. Priming was found following both the valid and invalid cues. This would suggest that the facilitation did not require directed attention. The provision of the dual-task required participants to maintain their gaze on the central fixation in order to perform the decision task. In this regard, the task set was to direct the eyes to the centre of the screen. Saccades toward the cued stimulus would not be compatible with the saccade programme (Koval et al., 2005).

It may be argued that the 100 ms duration of the prime may still allow some eye movement to occur. To investigate this, a further experiment was conducted where the prime duration was reduced to a minimum, and the prime image was masked. This would ensure that the prime occurred with minimal awareness and that any effects due to direction of gaze and saccadic eye movements were impeded.

## Experiment 6: Masked Priming

Experiment 6 examined the effect of shape priming using the same cueing procedure as used in Experiment 2 except that the prime duration and interval were reduced to 50 ms. Presented immediately prior to and following the prime was a noise mask. This would ensure that participants remained minimally aware of the prime. The trial sequence is illustrated in Figure 2. If priming occurred at a short duration and with a masked presentation, then this would provide evidence that the priming was occurring very early in the recognition process. It would also discredit interpretations that may suggest eye movements were a bias of the facilitatory effects reported.

In this study, the stimuli from Experiment 2 were reformed to include only the outline shape primes and neutral primes with the corresponding neutral and non-object trials. A pilot study found that at a 50 ms presentation they were unable to name the prime items that appeared between the noise mask. Participants were at chance level when they were asked to perform a category decision on the items. This confirmed that participants were aware that a prime had occurred but remained only minimally aware of the prime identity.

### Method

The method was the same as in previous experiments except where mentioned otherwise. There were 3 priming conditions—related shape primes, neutral primes, and unrelated shape primes. There were 2 Cue conditions, namely, Valid or Invalid Cues. The numbers of stimuli per condition and the proportion of valid: invalid trials were kept the same as the previous experiments. The prime was proceeded by a Gaussian noise mask for 10 ms, followed by a prime for 50 ms followed by a Gaussian Noise Mask of 10 ms. The target then appeared, and as in the previous experiments, participants were asked to make an object decision by keypress using their dominant hand. The trial sequence is illustrated in Figure 2.

#### Participants

The study group consisted of 20 participants (12 male, 8 female). from Kings Lynn Vision Plus Ltd., who volunteered to take part in the experiment. The ages ranged from 20 to 50 years (mean 40, *SD* = 9) and none had taken part in the previous experiments.

### Results

#### Reaction Times

The mean RTs are illustrated with the 95% CI in Figure 3. As in the previous experiments, data points of more than 3 *SD*s from each participant's mean were treated as errors (<1% of the total responses). There was a significant effect of Cue [*F*_(1,19)_ = 62.01, *p* = 0.0001] indicating that response times to targets following the valid cues (464 ms, *SD* = 31) were faster than those following invalid cues (487 ms, *SD* = 44). There was a significant effect of Priming [*F*_(2,19)_ = 43.36, *p* = 0.001]. Comparisons of means found a significant differences between the related vs. neutral (mean diff: 53.75, crit diff: 15.5, and *p* = 0.001) and related vs. unrelated (mean diff: 44.95, crit diff: 15.5, and *p* = 0.001) whereas the neutral vs. unrelated were not significantly different (mean diff: 8.77, crit diff: 15.5, and *p* = 0.16 NS). The interaction between Cue and Priming was not significant [*F*_(2,38)_ = 2.05, *p* = 0.14 NS].

#### Errors

The percentage errors were higher in the Neutral and Unrelated conditions (2.6 and 2.4%, respectively), compared to the Related condition (0.9%). Fewer errors were found following valid presentations (valid 1.6%, invalid 2.6%). The main effect of Cue approached significance [*F*_(1,19)_ = 3.19 *p* = 0.08]. There was a main effect of Priming [*F*_(2,19)_ = 16.15, *p* = 0.0001]. Comparisons of means showed that there were significant differences between all conditions related vs. neutral (mean diff: 0.32, crit diff: 0.3, and *p* = 0.01) and related vs. unrelated (mean diff: 0.7, crit diff: 0.3, and *p* = 0.001) neutral vs. unrelated were (mean diff: 0.4, crit diff: 0.3, and *p* = 0.004). The interaction between Cue and Priming was not significant [*F*_(2,38)_ = 1.93 *p* = 0.15].

### Discussion

Experiment 6 demonstrated the effects of shape priming from a similar whole object shape, even when the prime was masked, and the prime duration and inter-stimulus interval was reduced to 50 ms. This confirms that the priming effects reported in the previous experiments had the capacity to occur very early in the recognition process. Certainly, it would appear that the facilitation of shape is occurring before participants could identify the prime object. The locus of this effect is possibly a pre-attentive stage of processing. Although the prime presentation was brief, there is some debate as to the point in time in which pre-attentive processing becomes attentive processing. Nevertheless, the data demonstrate the priming of shape occurred with minimal attention and at a duration that could not easily be explained with claims regarding the use of eye movements between the prime and target.

It might be argued that the direction of gaze effects may have contributed and drawn attention toward an expected location however the accuracy data do not support this interpretation. Although there was a marginally higher accuracy following valid cues, this effect was not significant. Furthermore, significant priming occurred for both valid and invalid was cues. The brief duration of the masked prime demonstrates that with minimal awareness, the outline shape is still able to produce facilitation of the target, even when participants are unable to recognise an object.

One reason why global outline shapes may support object priming here is if the global shape is conveyed better through certain spatial frequency components when compared with component stimuli. Parker et al. (1996) and Bar (2003), amongst others, have argued that low spatial frequency representations of objects are derived rapidly and can serve to modulate the subsequent object recognition process. Possibly the proposed global, intermediate representations mediating priming here reflect the rapid assembly of a low spatial frequency description of the stimuli, which can serve to facilitate subsequent object recognition. This was evaluated in Experiment 7 the prime images were filtered so that either low or high spatial frequency components were available.

## Experiment 7: Spatial Frequency Filtering

Experiment 7 investigated whether differences in structural priming would occur when the images depict high and low spatial frequency components of the objects. High spatial frequency components of an image can provide information about stimulus features and their spatial arrangement within the image, whereas low spatial frequency components can convey shape and edge information in the absence of other detail. This experiment assessed which of these components was able to support structural priming.

### Method

Unless otherwise mentioned the method was the same as for Experiment 3. For each prime-target pair, a high spatial frequency and low spatial frequency prime were formed using the filtering function in Adobe Photoshop. A factorial design was used with Cue (valid and invalid) and Prime (whole object, HSF, LSF, and neutral) as within-participants factors. Examples of the stimuli are depicted in Figure 1 and the sequence of trials is illustrated in Figure 2. The numbers of stimuli per condition and the proportion of valid:invalid trials were kept the same as the previous experiments.

#### Participants

Twenty participants took part (11 men, 9 women). The average age was 47 years. The data from two participants were replaced because they made more than 50% errors overall. As in previous experiments, participants watched the screen and made an object decision to the targets through keypress.

### Results

#### Reaction Times

The mean correct RTs are illustrated in Figure 3. As in the previous experiments, data points of more than 3 *SD*s from each participant's mean were treated as errors (<1% of the total responses). The data were analysed with ANOVA. There were significant main effects of Cue [*F*_(1,19)_ = 25.44, *p* < 0.0001] and Prime [*F*_(3,57)_ = 1,135, *p* < 0.001]. RTs were faster on valid than invalid trials. RTs were also faster following whole object primes than neutral primes (mean diff: 40.05, crit diff: 24.43, and *p* < 0.05) and also for the whole object primes compared with the HSF and LSF (HSF: mean diff: −25.77, crit diff: 22.19, and *p* < 0.05; LSF: mean diff: −19, crit diff: 18.46, and *p* < 0.05). Differences between the neutral primes and HSF and LSF primes did not reach significance (HSF: mean diff: 14.32, crit diff: 18.46, and *p* > 0.05; LSF: mean diff: 21.08, crit diff: 22.19, *p* > 0.5. The interaction between Cue and Prime was not significant [*F*_(3,57)_ < 1, *p* = 0.4].

#### Errors

The error data were analysed with ANOVA. The percentage errors were higher in the Neutral conditions (7.1%) compared to the Related conditions (2.1%). The percentage errors were marginally higher following valid cues (3.9%) compared to invalid cues (3.3%). The effect of cue approached significance [*F*_(1,19)_ = 3.95, *p* = 0.06] and there was a significant effect of Prime [*F*_(3,57)_ = 7.16, *p* = 0.0004]. The interaction between cue and prime type was not reliable [*F*_(3,57)_ = 2.24 *p* > 0.05].

### Discussion

In Experiment 7, we only found reliable priming from the whole object primes. When the primes were subjected to a spatial frequency filtering the priming was reduced/eliminated. In respect of the RT data, the mean difference between normal primes and HSF primes was greater than normal primes and LSF primes, although these differences did not yield a reliable interaction between the effects of Cue and Prime.

## General Discussion

We examined structural priming from briefly presented primes onto object and non-object targets, for object decisions. Across all experiments, we established robust facilitatory effects of whole object primes on responses to targets when the primes were structurally but not conceptually related to the target (Experiment 1). These effects were equal in magnitude for object and non-object targets, indicating that the effects are not based on primes pre-activating stored representations for targets (Experiment 2). The effects did not depend on the presence of internal features in objects (Experiment 3), but it did require the outline shape to be present (Experiments 4). Fragmenting the external contour disrupted priming, even when full outline and fragmented contour stimuli had the same numbers of pixels (Experiment 4). The priming effect also occurred without directed or conscious awareness (Experiments 5 and 6). The priming effect was conveyed by low spatial frequency components from primes, but there was no benefit from high spatial frequency components alone (Experiment 7).

Biederman (1987a,b) has argued that humans recognise visual objects by identifying component parts known as “*geons.”* These geons are matched with representations stored in memory. However, contrary to Biederman's theory, the data presented in this paper appear to oppose the role of object parts in visual recognition and suggest that during the processing of the primes, some form of global intermediate representation was coded. These representations are sensitive to the outline shape of the stimulus but are not derived from a parts-based construction process.

Marr (1982) and Riddoch and Humphreys (2001) suggested that the perceptual system actively assembles information based on axis, lines, and edges. The current data suggest that contour provides an important foundation for visual object recognition, and this may form a key stage in image processing. This interpretation would concur with evidence from patients with brain damage (Warrington and Taylor, 1978; Solms et al., 1988; Lawson and Humphreys, 1998; Riddoch and Humphreys, 2001) and also with computational models such as that of Marr (1982) where contour and the axis of elongation form the foundation for the visual recognition process. The findings from the experimental manipulation of spatial frequency (Experiment 7) concur with findings from the neuropsychological case reported by Lawson and Humphreys (1999) and also concur with object-based theories of attention (Duncan, 1984; Duncan and Humphreys, 1989; Chen, 2012). This suggests that the intermediate representations mediating priming are coded pre-attentively and modulate target processing irrespective of whether the target falls at an attended or unattended location. This is in discordance with the evidence on parts-based priming, where attention to the prime may be necessary to generate the priming effect (Stankiewicz et al., 1998).

Alongside the evidence for structural priming, the current studies demonstrate that priming was always additive with the effects of attentional cueing reflecting whether primes and targets fell in the same or different locations. We assume that participants attended exogenously to the location of the briefly presented primes. Despite this, there was no change in the priming effect when targets were presented at an unattended spatial location (on invalid trials).

We propose that this global, intermediate representation is coded independently of a parts-based construction process, thereby supporting priming, even in the absence of parts-based object coding. Structural priming emerges when the intermediate representations of primes and targets overlap so that the representation of the prime boosts target processing. The argument for directly coded object representations, determined by low spatial frequency components in the image, fits with neuropsychological evidence that parts-based object recognition can break down whilst aspects of global object processing are preserved (see Riddoch et al., 2008; Lestou et al., 2014, for evidence). Moreover, Bar (2003) has also argued that object recognition involves the rapid extraction of coarse object descriptions, based on low spatial frequency components in the image, which are then fed back to modulate further object processing. The results of Experiment 7 are consistent with Bar's account–low spatial frequency information appears to mediate the perceptual process. Theoretically, what constitutes a local element, part or geon is a topic of continued debate. The definition may be a derivative of conjunctions and boundaries or relate to axis-based primitives (Schill et al., 2020; Wolfe, 2020). Although our account contradicts some “single process” approaches to object recognition (e.g., the parts-based “recognition by components” view of Biederman et al., 1999), it does concur with a hybrid account in which global shape representations are computed “on the fly” independently of feature coding (Stankiewicz et al., 1998).

We suggest that intermediate global representations of objects are coded pre-attentively but are insufficient to support parts-based priming. It will be of interest to assess whether the representations also only support view-specific but not view-invariant priming effects (Stankiewicz et al., 1998). It will also be of interest to explore the neural basis of these effects. Prior imaging studies suggest that some aspects of global form are coded through the parietal cortex and operate independently of the ventral visual stream (Lestou et al., 2014). Whether this is the case here awaits future experiments.

## Author's Note

This work was carried out at Oxford in association with Glyn Humphreys prior to his unexpected passing in 2016. A series of experiments provides evidence of an intermediate stage of representation in which objects are computed online. The effects were based on the overall outlines of the stimuli and low spatial frequency components, not on local object parts. The data indicate that the recognition process does not require the allocation of attention, with wholistic form providing a key stage of object recognition.

## Data Availability Statement

The raw data supporting the conclusions of this article will be made available by the authors, without undue reservation.

## Ethics Statement

The studies involving human participants were reviewed and approved by Prof. Glyn Humphreys, Department of Experimental Psychology University of Oxford. The patients/participants provided their written informed consent to participate in this study.

## Author Contributions

JH and GH contributed to conception and design of the study. JH wrote the first draft of the manuscript. All authors contributed to manuscript revision, read, and approved the submitted version.

## Funding

This work was supported by grants from the ESRC and ERC (Advanced Investigator award) to the GH.

## Conflict of Interest

The authors declare that the research was conducted in the absence of any commercial or financial relationships that could be construed as a potential conflict of interest.

## Publisher's Note

All claims expressed in this article are solely those of the authors and do not necessarily represent those of their affiliated organizations, or those of the publisher, the editors and the reviewers. Any product that may be evaluated in this article, or claim that may be made by its manufacturer, is not guaranteed or endorsed by the publisher.
